# Cumulative response of ecosystem carbon and nitrogen stocks to chronic CO_2_ exposure in a subtropical oak woodland

**DOI:** 10.1111/nph.12333

**Published:** 2013-05-30

**Authors:** Bruce A Hungate, Paul Dijkstra, Zhuoting Wu, Benjamin D Duval, Frank P Day, Dale W Johnson, J Patrick Megonigal, Alisha L P Brown, Jay L Garland

**Affiliations:** 1 Department of Biological Sciences and The Center for Ecosystem Science and Society, Northern Arizona UniversityFlagstaff, AZ, 86011, USA; 2 US Geological SurveyFlagstaff, AZ, 86001, USA; 3 US Dairy Forage Research Center, USDA-ARSMadison, WI, 53706, USA; 4 Department of Biological Sciences, Old Dominion UniversityNorfolk, VA, 23529, USA; 5 Department of Natural Resources and Environmental Science, University of NevadaReno, NV, 89557, USA; 6 Smithsonian Environmental Research CenterEdgewater, MD, 21037, USA; 7 Environmental Protection Agency, Microbiological and Chemical Exposure Assessment Research DivisionCincinnati, OH, 45268, USA

**Keywords:** carbon cycling, elevated CO_2_, global change, long-term experiment, nitrogen cycling, scrub oak, soil carbon, subtropical woodland

## Abstract

**Summary:**

- Rising atmospheric carbon dioxide (CO_2_) could alter the carbon (C) and nitrogen (N) content of ecosystems, yet the magnitude of these effects are not well known. We examined C and N budgets of a subtropical woodland after 11 yr of exposure to elevated CO_2_.
- We used open-top chambers to manipulate CO_2_ during regrowth after fire, and measured C, N and tracer ^15^N in ecosystem components throughout the experiment.
- Elevated CO_2_ increased plant C and tended to increase plant N but did not significantly increase whole-system C or N. Elevated CO_2_ increased soil microbial activity and labile soil C, but more slowly cycling soil C pools tended to decline. Recovery of a long-term ^15^N tracer indicated that CO_2_ exposure increased N losses and altered N distribution, with no effect on N inputs.
- Increased plant C accrual was accompanied by higher soil microbial activity and increased C losses from soil, yielding no statistically detectable effect of elevated CO_2_ on net ecosystem C uptake. These findings challenge the treatment of terrestrial ecosystems responses to elevated CO_2_ in current biogeochemical models, where the effect of elevated CO_2_ on ecosystem C balance is described as enhanced photosynthesis and plant growth with decomposition as a first-order response.

## Introduction

Many experiments have examined the responses of plant production and ecosystem carbon (C) balance to rising atmospheric CO_2_ (Reich *et al.*, 2006a; Norby & Zak, 2011). Results from these feature prominently in assessments of potential feedbacks between the biosphere and the changing atmosphere (Dolman *et al.*, 2010). Compared to responses of photosynthesis and plant growth to elevated CO_2_, the response of soil C is less well understood, because changes in soil C content are difficult to detect (Smith, 2004). Increased C in soil in response to elevated CO_2_ is sometimes found (Jastrow *et al.*, 2005; Iversen *et al.*, 2008, 2012), although more frequently there is no effect, whether because of low statistical power or the absence of an important effect is unclear (Hungate *et al.*, 2009). Ecosystem-scale inventories assessing C balance responses to elevated CO_2_ also often show no effect (Hungate *et al.*, 1997b; Gielen *et al.*, 2005; Gill *et al.*, 2006; Niklaus & Falloon, 2006; Adair *et al.*, 2009), although in aggregate some analyses suggest an effect is apparent (Luo *et al.*, 2006). Thus, global models projecting future C dynamics of the biosphere have strong support for the effects of CO_2_ on plant growth (Denman *et al.*, 2007), but less empirical support for assumed effects on total ecosystem C storage. Our first goal in this work was to construct a complete C inventory for a subtropical oak woodland after 11 yr of exposure to elevated CO_2_, to test whether the CO_2_ treatment altered total system C accumulation, and determine how any changes in C accumulation were distributed among plant and soil pools.

Total ecosystem C content is a function of plant growth and accumulation of plant biomass and detritus and also of C losses through microbial decomposition. Microbial decomposition is typically assumed to be a first-order process (Parton *et al.*, 1987), responding predictably and constantly to changes in substrate supply, and thus is not expected to respond to elevated CO_2_ independently of changes in substrate accumulation (Denman *et al.*, 2007). Challenging this idea, inputs of C to soil can stimulate mineralization of native soil organic matter (Lohnis, 1926; Broadbent & Norman, 1947; Broadbent & Bartholomew, 1949; Van Veen *et al.*, 1991), and increased atmospheric CO_2_ has been shown to promote microbial activity (Dieleman *et al.*, 2010) and even soil C loss (Hoosbeek, 2004; Trueman & Gonzalez-Meler, 2005; Carney *et al.*, 2007; Hagedorn *et al.*, 2008; Paterson *et al.*, 2008; Taneva & Gonzalez-Meler, 2008; Langley *et al.*, 2009; Trueman *et al.*, 2009; Drake *et al.*, 2011; Reid *et al.*, 2012). Thus, soil processes influence potential C accumulation in response to increasing atmospheric CO_2_, yet how and to what extent are not well understood. Our second goal in this work was to examine changes in soil microbial activity during the 11 yr of CO_2_ enrichment, and to test whether patterns of CO_2_ effects on soil microbial activity might help explain any effects (or lack of effects) of elevated CO_2_ on soil C stocks.

Carbon cycling in ecosystems is linked to cycles of other elements (Finzi *et al.*, 2011), such as nitrogen (N). Simulations of land carbon uptake using models with coupled N and C dynamics usually differ, and in many cases differ strongly, from those ignoring N (e.g. compare Cramer *et al.*, 2001 and Thornton *et al.*, 2007), because N limits plant growth and C storage (LeBauer & Treseder, 2008), and because N cycling is sensitive to environmental change (Galloway *et al.*, 2008). With N cycling included, simulations project smaller increases in terrestrial C storage in response to rising CO_2_, because N availability limits plant growth and its response to elevated CO_2_ (Thornton *et al.*, 2007; McMurtrie *et al.*, 2008; Sokolov *et al.*, 2008; Jain *et al.*, 2009; Wang & Houlton, 2009; Friedlingstein & Prentice, 2010; Zaehle *et al.*, 2010).

While model simulations bear out the importance of including N, these models do not necessarily demonstrate a consistent pattern of effect. Results differ in magnitude, direction and mechanism, suggesting that additional data and analyses are needed to evaluate conditions under which C–N coupling is important. For example, some simulations project only a modest limitation of terrestrial C uptake with coupled C–N interactions in the long term (at equilibrium), but strong effects of C–N interactions on the dynamics of C cycling and storage after disturbance (Gerber *et al.*, 2010). Although the models generally agree that including N limitation of plant production reduces the terrestrial C sink, the magnitude of this effect is highly variable (Arneth *et al.*, 2010). Experiments also indicate that C–N interactions are critical modulators of the long-term CO_2_ fertilization response, but different experiments provide support for different mechanisms underlying that modulation. In some cases, C–N interactions appear to constrain strongly the CO_2_ response (Reich *et al.*, 2006a,b; Norby *et al.*, 2010; Garten *et al.*, 2011), but in others, plants appear able to access the extra N needed to support the growth response (Johnson *et al.*, 2006; Drake *et al.*, 2011). Effects of CO_2_ concentration on microbial N transformations that influence the plant–soil distribution of N are extremely variable, with negative, positive and neutral effects observed for the same processes (Díaz *et al*., 1993; Zak *et al.*, 1993; Morgan *et al.*, 1994; Zanetti *et al.*, 1996; Hungate *et al.*, 1997a,c; Johnson *et al.*, 1997; Hofmockel & Schlesinger, 2007; van Groenigen *et al.*, 2011, 2012). Furthermore, other concomitant global environmental changes will modulate N constraints on C balance responses to elevated CO_2,_ including changes that alter N cycling directly, such as warming, altered precipitation and atmospheric N deposition, as well as indirect effects, such as changes in plant species composition. There is considerable debate as to the magnitude of the impact of such effects on ecosystem C sequestration, however (Jenkinson *et al.*, 1999; Nadelhoffer *et al.*, 1999; Arneth *et al.*, 2010). Thus, both model simulations and data can be invoked to support N cycling constraining, increasing, or having little effect on the terrestrial C sink. Our third goal in this research was to compare C and N inventories in response to 11 yr of CO_2_ exposure in a subtropical woodland, in order to test how rising CO_2_ affects these elements in concert.

One of the challenges in investigating C–N interactions in ecosystem experiments is that the timescale of measurements of N cycling rates is typically far shorter than the timescale of N cycling processes that influence ecosystem responses. Elevated CO_2_ can alter multiple processes within the soil N cycle simultaneously, with strong temporal dynamics, and with opposing impacts on plant N availability, making it very difficult to extrapolate short-term measurements to long-term effects. Following an isotope tracer over multiple years can help overcome this challenge. ^15^N tracers reflect short-term effects on N cycling processes and integrate these into long-term effects on ^15^N distribution among plant and soil components within the system. Because the ^15^N is added in labile form, losses of added ^15^N will be relatively larger than losses of total ecosystem N, so can be detected with greater sensitivity. Our fourth goal in this research was to use a long-term ^15^N tracer to characterize changes in N distribution and N losses in response to elevated CO_2_.

Here, we report a whole system inventory of the C and N content of a scrub-oak ecosystem after 11 yr of experimental CO_2_ exposure. We also show how soil microbial activity responded to chronic CO_2_ exposure. We also report recovery and distribution of a ^15^N tracer applied early in the experiment, in order to assess how elevated CO_2_ alters the system-level distribution of labile N over the timescale of a decade.

## Materials and Methods

The scrub-oak experiment occurred at the Merritt Island National Wildlife refuge on the east coast of Florida, USA (28°38′N, 80°42′W). After controlled burning, 16 open-top chambers were established over the regrowing vegetation, each covering 9.42 m^2^ ground area, with 8 chambers receiving ambient air and 8 receiving ambient air + 350 ppm V CO_2_ (referred to as the ‘elevated CO_2_' treatment). A large blower circulated air through each chamber at a rate of 24–30 m^3^ min^−1^, replacing the chamber air volume 1.3–1.6 times min^−1^ (Dijkstra *et al.*, 2002). The chambers increased air temperature and vapor pressure deficit while decreasing light (Dore *et al.*, 2003), micro-environmental effects that did not significantly alter growth or species composition (Seiler *et al.*, 2009). The experiment began in May 1996 and was maintained until June 2007.

In June–July 2007, all aboveground material was harvested from the chambers (see Seiler *et al.*, 2009), and roots and soils were collected using multiple cores in each chamber (see Day *et al.*, 2013). For aboveground biomass, all shoots were cut at the base of the stem, weighed immediately, and subsampled for the determination of water content and elemental analysis of leaves and stems. Ten surface cores (0–10 cm) and five deep cores were collected from each plot at 10 cm increments; all cores were 7 cm diameter. Core depth varied among plots from 2 to 3 m due to differences in the depth to the water table and the spodic (B_h_) horizon. For purposes of the element inventory conducted here, depth increments were combined into 0–10, 10–30, 30–60 and 60–100 cm. Samples were hand-picked to remove large roots, and subsamples separated into coarse particulate organic matter, roots and mineral soil. Belowground biomass was also sampled indirectly using ground-penetrating radar (Stover *et al.*, 2007, Day *et al.*, 2013). Material on the forest floor was gathered from 1/8th of each plot by hand, collecting until no visibly identifiable plant fragments remained. Material was dried, sifted to remove adhering sand, and weighed.

We used a combination of density and biological fractionations to estimate soil carbon (C) pools of varying turnover rates. We used incubations to estimate labile and active soil C pools (and, by difference residual C), using the technique of Nadelhoffer (1990). We measured CO_2_ production from laboratory incubations, combining short-term incubations of soils immediately after collection (McKinley *et al.*, 2009) with 541-d incubations conducted in the lab at Northern Arizona University. We used density fractionations as described previously (Hungate *et al.*, 2006; Carney *et al.*, 2007), separating light (< 1.5 g cm^−3^), medium (1.5–1.8 g cm^−3^), heavy (1.8–2.2 g cm^−3^) and residual (> 2.2 g cm^−3^) organic matter fractions. Total soil C, N, ^15^N and ^13^C were also measured on bulk samples collected from the cores. Our fractionation analysis focused on soils from the 0–60 cm depths. For bulk soil analyses where we measure total C, N and ^15^N, we present the data to 1 m to correspond with the depth of the root biomass inventory.

We measured microbial biomass using the chloroform-fumigation extraction method (Vance *et al.*, 1987) in mineral soil (0–15 cm) sampled in July 1997; June, July, September and December 1998; September 1999; and May 2004. Soil subsamples (20–25 g at field moisture content) were extracted in 75 ml 0.5 M K_2_SO_4_ before and after 24-h fumigation with ethanol-free chloroform. The K_2_SO_4_ extracts were dehydrated in a forced-air drying oven at 60°C, the salts ground in a mortar and pestle, and the resulting powder analyzed for C, N, δ^15^N and δ^13^C on a CE 2100 elemental analyzer coupled to a Thermo DeltaPLUS-XL isotope-ratio mass spectrometer (http://www.isotope.nau.edu). Microbial biomass was calculated as the difference in mass (of C, N, ^13^C or ^15^N) between fumigated and nonfumigated samples, divided by 0.54 to correct for extraction efficiency (Vance *et al.*, 1987). For samples collected after the ^15^N tracer application (June 1998), we also measured the ^15^N content of mineral soil (0–15 cm depth). After milling, soil N and ^15^N contents were determined as described above.

The CO_2_ added to the elevated-CO_2_ treated plots was depleted in ^13^C. We used a two-member mixing model to determine mineral soil C derived from new photosynthate (Leavitt *et al.*, 1994; Hungate *et al.*, 1996). Stem tissue produced in the elevated CO_2_ treatment (δ^13^C_S,E_) provided an integrative measure of the δ^13^C value of new photosynthate (average across five sampling dates, -42.6 ± 0.3 ‰). However, because mineral soil (δ^13^C_M,A_) and stem δ^13^C (δ^13^C_S,A_) differed in the ambient *C*_a_ treatment, we calculated the δ^13^C signature of new carbon (δ ^13^C_new_) as: Eqn 1
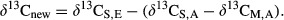


The δ ^13^C of the mineral soil in the ambient CO_2_ treatment was used as the end member for organic matter fixed before the experiment began. Carbon, N, ^15^N, and ^13^C were determined for all plant and soil components using coupled Dumas combustion isotope-ratio mass spectrometry (Carlo-Erba elemental analyzer and Finnigan Delta-V mass spectrometer) at the Colorado Plateau Stable Isotope Laboratory (www.isotope.nau.edu).

For testing soil microbial activity, we collected soil and litter samples in May through July of 2004, after 8 yr of CO_2_ treatment. Soil sampling, preparation of microbial inocula, carbon and nutrient amendments, and incubation conditions are described in Brown *et al*. (2009). Carbon substrates included glucose and hot-water extracts of roots and leaf litter collected from the ambient and elevated CO_2_ treatments. Microbial inocula from litter, rhizosphere and bulk soil communities were also prepared from the two CO_2_ treatments. We used the BD-oxy system (BD Oxygen Biosensor System, BD Biosciences, Bedford, MA, USA (Garland *et al.*, 2003; Väisänen *et al*., 2005; Zabaloy *et al.*, 2008) to evaluate microbial respiration. The system uses a fluorophore that fluoresces as O_2_ is consumed during the 48 h incubation. Normalized relative fluorescence was calculated as relative fluorescence after 48 h normalized by dividing by relative fluorescence after 1 h. The response to substrate addition was calculated as:
Eqn 2
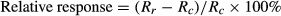


(*R*_*c*_, normalized relative fluorescence in the absence of resource addition; *R*_*r*_, normalized relative fluorescence with the added resource. Brown *et al*. (2009) present data from the ambient CO_2_ treatment; here, we expand on this past analysis to evaluate responses of microbial respiration to elevated CO_2_. We used ANOVA to test for effects of habitat (rhizosphere, litter or bulk soil), inoculum source (ambient or elevated CO_2_), substrate source (ambient or elevated CO_2_), substrate type (litter or root), N, and P. We used a separate ANOVA to test compare responses to the addition of glucose vs natural substrates extracted from roots and litter. Where appropriate, ANOVAs were designed as split-plots, to account for the nonindependence of inocula collected from individual experimental plots subject to multiple combinations of resource treatments in the BD-Oxy assay.

We used resampling to infer the effects and estimate the magnitude of the elevated CO_2_ treatment on ecosystem C and N pools and recovery of tracer ^15^N. We estimated 5% and 95% confidence limits for the difference in means between elevated and ambient CO_2_ treatments, using 1000 samples with replacement (*n *=* *8 for each treatment).

## Results

Elevated CO_2_ increased plant biomass, including the mass of C (g C m^−2^) in leaves, stems and coarse roots, and the total mass of C in plants (Table 1). The mass of C in fine roots was not significantly affected by the elevated CO_2_ treatment at the final harvest (Table 1), although fine roots did exhibit significant increases at other times during the experiment (Day *et al.*, 2013). On average, plant C accumulation by the end of the experiment was 71.5 g C m^−2^ yr^−1^ higher in elevated compared to ambient CO_2_, roughly equally distributed aboveground (37.5 g m^−2^ yr^−1^) and belowground (33.5 g m^−2^ yr^−1^). The C content of the litter layer, coarse particulate organic matter, total mineral soil C, and the light and medium density fractions did not significantly respond to the CO_2_ treatment, whereas the heavy density soil C pool significantly declined. Elevated CO_2_ had no effect on soil C in the spodic horizon, with no significant effect on total mineral soil C, or on the light, medium and heavy density fractions (Table 2); thus, C in the deep soil was also insensitive to the CO_2_ treatment. In general, increased mass of plant C caused by elevated CO_2_ did not translate to increased C storage in other ecosystem reservoirs (Table 1).

**Table 1:** Inventory of carbon after 11 yr exposure to increased atmospheric CO_2_ in a subtropical oak woodland^a^


Carbon (g C m^−2^)	Ambient	Elevated	Effect	5% & 95% CLs
Aboveground	624.5 ± 54.6	1043.0 ± 77.5	418.5	(274.8 to 556.9)
Oak leaves	212.2 ± 22.3	318.4 ± 29.6	106.2	(47.6 to 157)
Oak stems	347.1 ± 34.2	621.6 ± 60.8	274.5	(164.8 to 374.2)
Other species	38.3 ± 10.7	63.1 ± 10.2	24.7	(1.7 to 47.4)
Standing dead	26.9 ± 8.4	39.8 ± 13.8	13.0	(−9.7 to 39.5)
Litter layer	332 ± 41.2	368.1 ± 42.4	36.1	(−57.9 to 127.7)
Roots	2886.7 ± 90.2	3261.3 ± 174.6	374.6	(73.6 to 674.5)
Fine roots	909.4 ± 62.8	803.9 ± 43.3	−105.5	(−226.8 to 9.7)
Coarse roots	1977.3 ± 102.8	2457.4 ± 177.7	480.1	(168.9 to 790.0)
Plant	3511.2 ± 102.0	4304.3 ± 221.3	793.1	(437.4 to 1172.7)
CPOM (0–100 cm)	1406.5 ± 386.4	1168.5 ± 272.1	−238.0	(−957 to 354.4)
Soil (0–100 cm)	5513.1 ± 411.5	5025.6 ± 647.4	−487.5	(−1456.5 to 636.8)
Light, 0–60 cm	2534.7 ± 260.2	2394.4 ± 333.3	−140.4	(−746.8 to 473.2)
0–10 cm	1530.9 ± 284.8	1415.8 ± 316.8	−115.0	(−760.1 to 565.5)
10–30 cm	480.2 ± 94.5	331.2 ± 36.5	−149.1	(−297.9 to 6.7)
30–60 cm	523.7 ± 149.9	647.3 ± 169.2	123.7	(−214.9 to 474.5)
Medium, 0–60 cm	1306.3 ± 302	1208.4 ± 177.3	−97.9	(−633.8 to 380.3)
0–10 cm	660.3 ± 115.3	560.7 ± 108.2	−99.6	(−346.4 to 158.9)
10–30 cm	370.9 ± 109.2	341.5 ± 55.2	−29.4	(−222.4 to 147.1)
30–60 cm	275 ± 157.8	306.2 ± 88.6	31.1	(−267.6 to 289.2)
Heavy, 0–60 cm	706.3 ± 120.5	396 ± 92.1	−310.4	(−553.2 to −86.0)
0–10 cm	110.9 ± 27	81.2 ± 19.9	−29.7	(−81.7 to 22.0)
10–30 cm	148 ± 23.7	83.5 ± 30.6	−64.6	(−122.9 to 2.0)
30–60 cm	447.4 ± 107.6	231.3 ± 87.2	−216.1	(−402.7 to −3.3)
Residual, 0–60 cm	965.8 ± 1026.9	1026.9 ± 330.9	61.1	(−782.3 to 925.2)
Soil (60–100 cm)	1547.0 ± 129.3	1877.6 ± 359.8	330.7	(−274.4 to 925.4)
Total ecosystem	12309.8 ± 582.1	12744.1 ± 444.3	434.4	(−723 to 1529.9)

a Values are means ± SE of the mean for the Ambient and Elevated CO_2_ treatments, the Effect of the CO_2_ treatment (E–A), and the bootstrapped 5% and 95% CLs (confidence limits) for the treatment effect. CPOM, coarse particulate organic matter. Soil fractions are density fractions, including light (< 1.5 g cm^−3^), medium (1.5–1.8 g cm^−3^), heavy (1.8–2.2 g cm^−3^) and residual (calculated as total soil total minus the sum of measured density fractions).

**Table 2:** Soil carbon (C) in the spodic horizon of the subtropical oak woodland

	Ambient	Elevated	*P*-value	Ambient	Elevated	*P*-value
	%C			δ^13^C		
Total C	0.77 ± 0.10	0.60 ± 0.15	0.383	−25.6 ± 0.1	−25.1 ± 0.3	0.157
Light	18.3 ± 2.8	11.9 ± 1.2	0.151	−25.3 ± 0.1	−25.3 ± 0.1	0.943
Medium	14.2 ± 3.8	9.4 ± 2.0	0.288	−25.6 ± 0.2	−25.3 ± 0.1	0.178
Heavy	13.2 ± 2.3	12.2 ± 1.0	0.710	−25.6 ± 0.1	−25.2 ± 0.2	0.116

Elevated CO_2_ increased the N content of plants aboveground (Table 3), but the N contents of coarse and fine roots did not respond to elevated CO_2_, yielding no effect on total plant N. The N content of most soil fractions was not significantly altered by elevated CO_2_, except the medium density fraction at 30–60 cm, which increased, and the light fraction at 10–30 cm, which declined. Increased C in plant pools with only small changes in N means higher C to N ratios. Higher C to N ratios under elevated CO_2_ were observed for leaves, coarse roots and the sum of all plant parts; elevated CO_2_ also increased the C to N ratio of the litter layer (Table 4). Elevated CO_2_ did not increase the C to N ratio of any soil pool; the only soil pool to respond – the heavy density fraction – actually declined in C to N ratio. Changes in plant and soil C to N ratios were compensatory, such that elevated CO_2_ had no effect on the C to N ratio of the plant–soil system to 1 m depth.

**Table 3:** Inventory of ecosystem nitrogen (g N m^−2^) after 11 yr exposure to increased atmospheric CO_2_ in a subtropical oak woodland^a^

	Ambient	Elevated	Effect	5% & 95% CLs
Aboveground	8.4 ± 0.8	13.1 ± 0.9	4.7	(3.0 to 6.3)
Oak leaves	4.7 ± 0.6	6.6 ± 0.6	1.9	(0.5 to 3)
Oak stems	3.0 ± 0.3	5.1 ± 0.5	2.2	(1.2 to 3.1)
Other species	0.5 ± 0.1	1.1 ± 0.2	0.5	(0.2 to 0.9)
Standing dead	0.2 ± 0.1	0.3 ± 0.1	0.1	(−0.1 to 0.3)
Litter layer	5.7 ± 0.7	6.0 ± 0.9	0.3	(−1.4 to 2.2)
Roots	29.3 ± 1.8	27.8 ± 2.5	−1.4	(−6.4 to 3.1)
Fine roots	8.3 ± 0.8	7.3 ± 0.9	−1.0	(−2.8 to 0.9)
Coarse roots	21.0 ± 1.2	20.5 ± 2.5	−0.4	(−4.6 to 4.2)
Plant	37.7 ± 1.8	41.0 ± 2.9	3.3	(−1.8 to 8.3)
CPOM (0–100 cm)	20.7 ± 5.7	15.2 ± 3.5	−5.4	(−15 to 3.0)
Soil (0–100 cm)	159.5 ± 15.0	145.4 ± 17.5	−14.2	(−44.2 to 15.9)
Light, 0–60 cm	55.9 ± 7.0	54.9 ± 8.9	−1.0	(−19 to 16.6)
0–10 cm	37.2 ± 7.6	37.6 ± 8.9	0.4	(−17.9 to 18.5)
10–30 cm	9.6 ± 1.8	6.5 ± 0.7	−3.1	(−5.8 to −0.1)
30–60 cm	9.1 ± 2.8	10.8 ± 2.1	1.7	(−4.2 to 6.4)
Medium, 0–60 cm	30.7 ± 5.5	30.9 ± 4.1	0.2	(−10.9 to 11)
0–10 cm	18.8 ± 3.4	15.4 ± 3.0	−3.4	(−10.4 to 3.5)
10–30 cm	7.9 ± 2.0	7.0 ± 1.0	−0.9	(−4.3 to 2.3)
30–60 cm	4.0 ± 1.5	8.5 ± 2.6	4.6	(0.4 to 9.3)
Heavy, 0–60 cm	17.3 ± 2.1	15.6 ± 4.9	−1.7	(−9.2 to 6.8)
0–10 cm	3.4 ± 0.8	2.4 ± 0.6	−1.0	(−2.7 to 0.5)
10–30 cm	4.1 ± 0.8	2.5 ± 1.1	−1.6	(−3.3 to 0.5)
30–60 cm	9.8 ± 1.5	10.7 ± 4.4	0.9	(−6 to 8.9)
Residual, 0–60 cm	55.6 ± 44.0	12.3 ± 13.5	−43.3	(−40 to 17.3)
Soil (60–100 cm)	51.3 ± 3.2	55.3 ± 8.8	4.1	(−10.8 to 18.2)
Total ecosystem	274.8 ± 10.9	262.9 ± 13.9	−12.0	(−38.1 to 18.9)

a Values are means ± SE of the mean for the Ambient and Elevated CO_2_ treatments, the Effect of the CO_2_ treatment (E–A), and the bootstrapped 5% and 95% CLs (confidence limits) for the treatment effect. CPOM, coarse particulate organic matter. Soil fractions are density fractions, including light (<1.5 g cm^−3^), medium (1.5–1.8 g cm^−3^), heavy (1.8–2.2 g cm^−3^) and residual (calculated as total soil total minus the sum of measured density fractions).

**Table 4:** Carbon to nitrogen ratios (g : g) in ecosystem components after 11 yr of experimental exposure of a subtropical woodland to increased atmospheric CO_2_^a^

	Ambient	Elevated	Effect	CI
Aboveground	74.7 ± 2.2	79.3 ± 2.1	4.6	(0.3 to 9.1)
Oak leaves	45.8 ± 1.0	48.3 ± 1.0	2.5	(0.2 to 4.9)
Oak stems	120.6 ± 7.9	121.2 ± 7.2	0.6	(−13 to 14.5)
Other species	71.9 ± 6.6	60.1 ± 7.4	−11.8	(−23.7 to −1.3)
Standing dead	108.5 ± 3.8	113.4 ± 4.8	4.9	(−4.5 to 13.8)
Litter layer	58.6 ± 1.5	64.2 ± 1.9	5.6	(0.7 to 10.6)
Roots	101.3 ± 7.4	122.0 ± 7.5	20.7	(2.3 to 39.7)
Fine roots	114.0 ± 9.6	116.3 ± 9.8	2.2	(−16.8 to 23.6)
Coarse roots	97.0 ± 8.1	128.9 ± 8.1	32.0	(7.1 to 59.3)
Plants	94.6 ± 5.3	106.9 ± 5.6	12.4	(0.8 to 23.4)
CPOM (0–100 cm)	71.1 ± 9.1	76.2 ± 8.7	5.1	(−10.6 to 17.9)
Soil (0–100 cm)	36.8 ± 2.2	37.8 ± 2.0	0.9	(−3 to 4.6)
Light, 0–60 cm	46.8 ± 3.9	45.0 ± 1.4	−1.8	(−9.1 to 5.1)
0–10 cm	43.7 ± 6.9	38.8 ± 0.8	−4.9	(−17.9 to 3.7)
10–30 cm	50.4 ± 2.5	51.9 ± 2.3	1.5	(−4.5 to 7.4)
30–60 cm	64.5 ± 9.7	60.8 ± 9.5	−3.7	(−21.8 to 11.2)
Medium, 0–60 cm	41.2 ± 3.0	39.5 ± 3.0	−1.7	(−8.2 to 4.1)
0–10 cm	35.4 ± 0.6	37.2 ± 0.7	1.8	(−0.3 to 4.4)
10–30 cm	45.6 ± 2.9	48.2 ± 2.9	2.7	(−3.4 to 8.8)
30–60 cm	53.2 ± 8.3	44.9 ± 8.6	−8.3	(−26.8 to 7.7)
Heavy, 0–60 cm	40.2 ± 4.2	29.4 ± 5.1	−10.7	(−18.5 to −3.5)
0–10 cm	32.8 ± 1.4	33.7 ± 1.4	0.9	(−1.6 to 3.6)
10–30 cm	41.2 ± 5.1	38.4 ± 5.1	−2.9	(−10.8 to 5.3)
30–60 cm	45.5 ± 10.0	27.7 ± 10.6	−17.8	(−36.6 to −3.4)
Residual, 0–60 cm	30.1 ± 1.4	33.2 ± 1.5	3.1	(−0.4 to 6.8)
Soil (60–100 cm)	31.6 ± 9.3	26.0 ± 9.4	−5.5	(−24.1 to 10.4)
Total ecosystem	45.0 ± 2.1	48.9 ± 1.9	3.9	(−0.1 to 8.1)

a Values are means ± SE of the mean for the Ambient and Elevated CO_2_ treatments, the Effect of the CO_2_ treatment (E–A), and the bootstrapped 5% and 95% CLs (confidence limits) for the treatment effect. CPOM, coarse particulate organic matter. Soil fractions are density fractions, including light (<1.5 g cm^−3^), medium (1.5–1.8 g cm^−3^), heavy (1.8–2.2 g cm^−3^) and residual (calculated as total soil total minus the sum of measured density fractions).

Elevated CO_2_ increased recovery of tracer ^15^N in aboveground plant tissues, but reduced recovery in coarse roots, in the soil light fraction at 10–30 cm depth, and in the soil residual fraction at 0–60 cm (Table 5). Together, these changes resulted in a significant decline in whole-system ^15^N recovery under elevated CO_2_. Elevated CO_2_ reduced the δ^15^N of plant tissue (weighted average of all plant parts), a dilution of the added ^15^N tracer with unlabeled ^15^N. This pattern indicates that elevated CO_2_ increased plant access to N, either through new N inputs or redistribution from existing ecosystem N reservoirs. But, because total plant N did not respond to elevated CO_2_, the increase in inputs of new N to plants were matched by N losses from plants, such that CO_2_ enhanced N turnover through the plant system. In contrast to plant δ^15^N, the δ^15^N of soils did not change with elevated CO_2_, nor was whole-system δ^15^N affected (Table 6).

**Table 5:** Inventory of tracer ^15^N (mg excess ^15^N m^−2^) after 11 yr exposure to increased atmospheric CO_2_ and 9 yr of integration of the added ^15^N tracer in a subtropical oak woodland^a^

	Ambient	Elevated	Effect	5% & 95% CLs
Aboveground	2.8 ± 0.4	3.7 ± 0.4	0.9	(0.1 to 1.8)
Oak leaves	1.6 ± 0.2	1.8 ± 0.3	0.2	(−0.3 to 0.7)
Oak stems	1 ± 0.2	1.6 ± 0.2	0.6	(0.2 to 0.9)
Other species	0.1 ± 0	0.2 ± 0	0.1	(0 to 0.2)
Standing dead	0.1 ± 0	0.1 ± 0	0.0	(0 to 0.1)
Litter layer	2 ± 0.3	2 ± 0.3	0.0	(−0.8 to 0.8)
Roots	7.7 ± 1.2	4.5 ± 0.6	−3.3	(−5.2 to −1.4)
Fine roots	2.1 ± 0.4	1.4 ± 0.2	−0.7	(−1.5 to 0)
Coarse roots	5.6 ± 1.1	3.1 ± 0.6	−2.6	(−4.5 to −0.7)
Plant	10.5 ± 1.0	8.2 ± 0.8	−2.4	(−4.2 to −0.4)
CPOM (0–100 cm)	0.6 ± 0.1	0.7 ± 0.1	0.1	(−0.2 to 0.3)
Soil (0–100 cm)	83.7 ± 16.4	59.2 ± 11.4	−24.5	(−53 to 3.7)
Light, 0–60 cm	28.5 ± 4	29.2 ± 4.8	0.7	(−8.9 to 10)
0–10 cm	21.6 ± 4.4	24 ± 5	2.4	(−8 to 12.8)
10–30 cm	4 ± 0.7	2.2 ± 0.2	−1.8	(−2.9 to −0.7)
30–60 cm	2.9 ± 0.9	3.1 ± 0.7	0.1	(−1.8 to 1.8)
Medium, 0–60 cm	15.2 ± 2.9	14.9 ± 2.4	−0.3	(−5.6 to 5.6)
0–10 cm	11.2 ± 2.1	10.1 ± 2.2	−1.2	(−6 to 3.7)
10–30 cm	2.8 ± 0.7	2.5 ± 0.4	−0.3	(−1.5 to 0.8)
30–60 cm	1.1 ± 0.4	2.3 ± 0.8	1.2	(−0.1 to 2.6)
Heavy, 0–60 cm	5.1 ± 0.8	4.3 ± 1.1	−0.8	(−2.7 to 1.3)
0–10 cm	1.9 ± 0.5	1.5 ± 0.4	−0.3	(−1.3 to 0.7)
10–30 cm	1.2 ± 0.2	0.8 ± 0.4	−0.4	(−1 to 0.3)
30–60 cm	2.1 ± 0.3	2 ± 0.8	−0.1	(−1.5 to 1.3)
Residual, 0–60 cm	34.8 ± 10.8	14.2 ± 9.5	−20.7	(−51.7 to −0.8)
Soil (60–100 cm)	5.8 ± 1.1	6.4 ± 1.7	0.6	(−2.5 to 4)
Total ecosystem	102.6 ± 15.7	76.4 ± 9.0	−26.2	(−55.1 to −0.8)

a Values are means ± SE of the mean for the Ambient and Elevated CO_2_ treatments, the Effect of the CO_2_ treatment (E–A), and the bootstrapped 5% and 95% CLs (confidence limits) for the treatment effect. CPOM, coarse particulate organic matter. Soil fractions are density fractions, including light (<1.5 g cm^−3^), medium (1.5–1.8 g cm^−3^), heavy (1.8–2.2 g cm^−3^) and residual (calculated as total soil total minus the sum of measured density fractions).

**Table 6:** δ^15^N signatures (mean ± SEM) of plant, soil and whole system N at the final harvest in July 2007

	Ambient CO_2_	Elevated CO_2_	*P*-value*
Plant	76.3 ± 5.4	55.0 ± 4.2	0.008
Soil	103.7 ± 13.9	85.4 ± 11.6	0.422
System	100.4 ± 11.6	80.8 ± 10.0	0.317

* *P-*values are for one-way ANOVAs testing the effect of elevated CO_2_.

**Fig 1:**
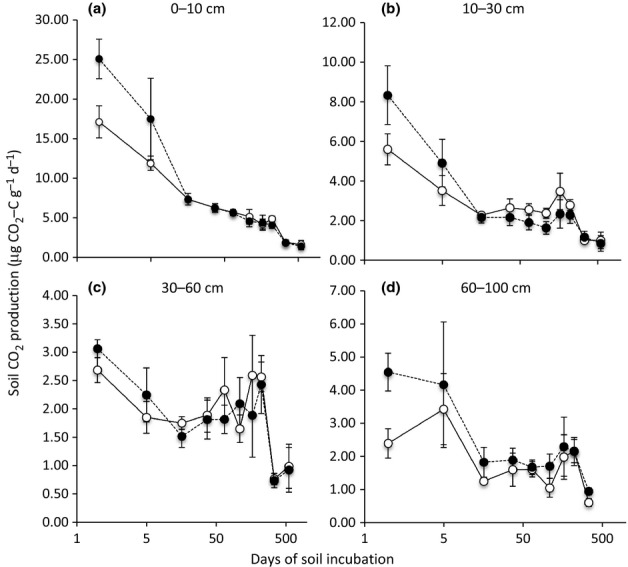
CO_2_ production during soil incuba-tions for four soil depths (a, 0–10 cm; b, 10–30 cm; c, 30–60 cm; d, 6–100 cm) in a subtropical oak woodland exposed to 11 yr of increased CO_2_. Ambient CO_2_, open circles; elevated CO_2_, closed circles. Bars show ± 2 SEM.

**Fig 2:**
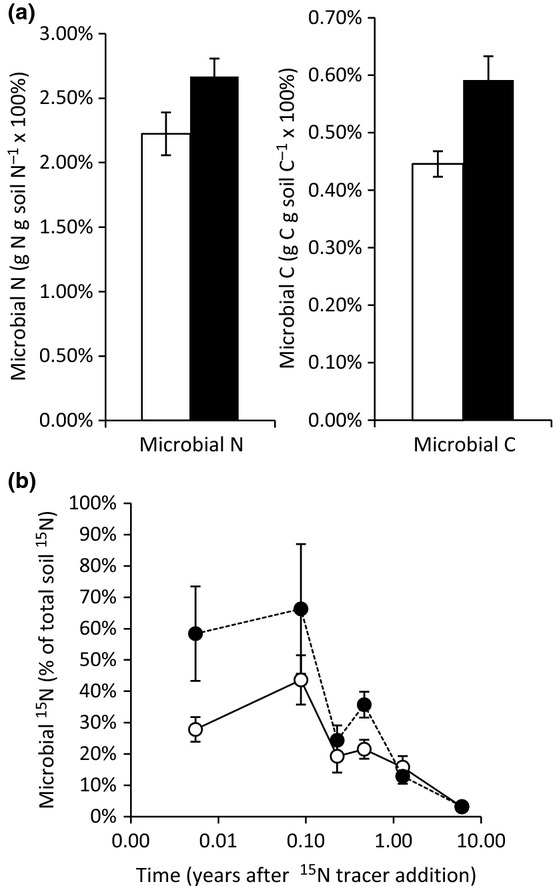
(a) Soil microbial biomass nitrogen (N) and carbon (C) from the ambient (open bars) and elevated (closed bars) CO_2_ treated plots. Microbial C and N (as a proportion of total soil C and N) are shown as means across seven sample dates spanning 1997 to 2004, years 2–9 of CO_2_ exposure. (b) Tracer ^15^N in the microbial biomass (as a proportion of tracer ^15^N in total soil) over time after label addition (log scale). Bars show ± 2 SEM.

**Fig 3:**
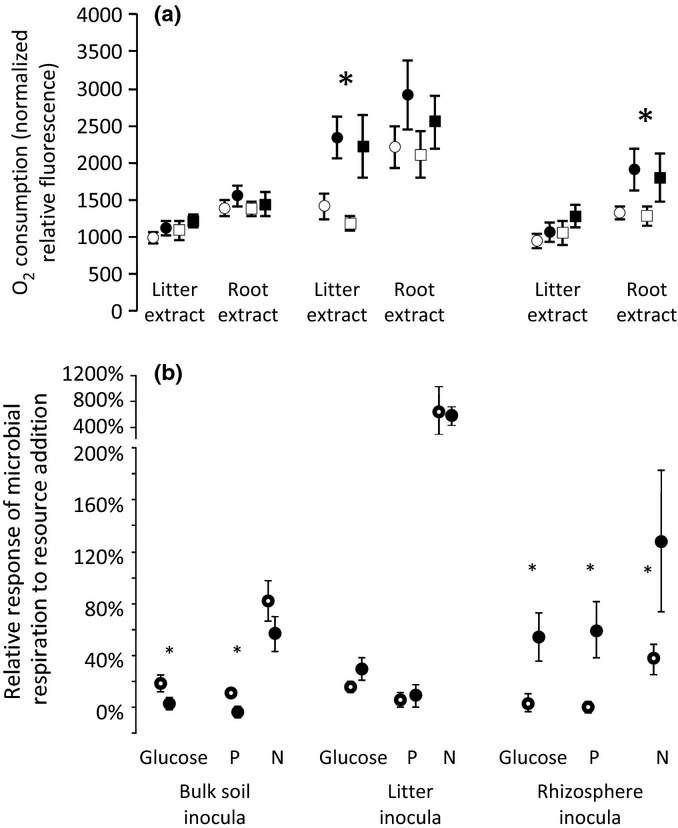
(a) Total respiration (O_2_ consumption, expressed as normalized relative fluorescence) of microbial inocula from three soil habitats (bulk soil, litter, rhizosphere) on extracts of litter and roots. Circles, inocula from ambient CO_2_; squares, from elevated CO_2_. Open symbols, substrates produced in the ambient CO_2_ treatment; closed symbols, substrates produced in the elevated CO_2_ treatment. Significant differences between substrates produced under ambient and elevated CO_2_ conditions (two-way ANOVAs, effect of substrate origin); *, *P *<* *0.050. (b) The relative responses of microbial respiration to single resource additions (glucose, N, or P) for microbial inocula from the bulk soil, litter and rhizosphere communities in the ambient (open circles) and elevated (closed circles) CO_2_ treatments. *Significant differences in resource limitation for individual comparisons (*t*-tests) of inocula from the ambient and elevated CO_2_ treatments. For full statistical results, see Supporting Information Tables S1 and S2. Bars show ± 2 SEM.

While elevated CO_2_ did not alter total ecosystem C, and effects on soil C were either nil or negative, several results indicate that elevated CO_2_ increased soil microbial activity. Elevated CO_2_ increased C mineralization in laboratory incubations, particularly for the first 24 h after collection in the field (Fig. 1, and see McKinley *et al.*, 2009), indicating a larger and more rapidly cycling labile soil C pool. Elevated CO_2_ also increased the proportion of soil organic matter that occurred in the soil microbial biomass: averaged across seven sample dates from 1997 to 2004, more soil C, N and ^15^N was contained in the soil microbial biomass in the elevated CO_2_ treatment (*P *=* *0.012 for C, *P *=* *0.096 for N, and *P *=* *0.049 for ^15^N; Fig. 2). When common inocula were presented with the labile substrates produced by leaves and roots, substrates produced in the elevated CO_2_ treatment were respired more completely than substrates from the same sources in the ambient CO_2_ treatment (Fig. 3a), indicating that the substrates produced in the high-CO_2_ environment were more susceptible to microbial decay. For the litter and rhizosphere microbial communities, microbial inocula from the elevated CO_2_ treatment consumed more O_2_ than inocula collected from the ambient CO_2_ treatment when presented with a common C substrate (Fig. 3b). Glucose induced a greater response in bulk soil inoculum from the ambient treatment (Fig. 3b), which may reflect CO_2_-depletion of available soil C susceptible to priming (Brown *et al.*, 2009).

**Fig 4:**
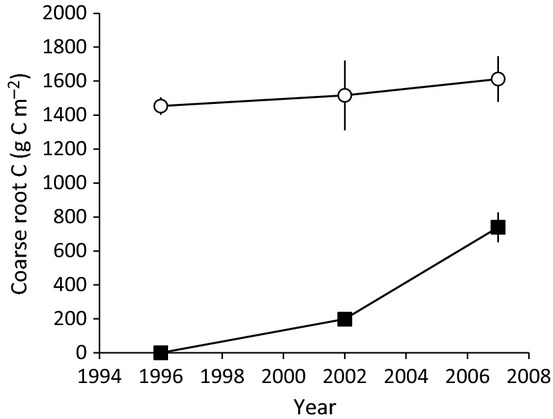
Coarse root carbon (C) over time in the scrub-oak experiment, showing ‘old’ (open circles) and ‘new’ (closed squares) carbon for the elevated CO_2_ plots, where new is defined as carrying a ^13^C isotopic signature of the CO_2_ added to the elevated CO_2_ plots. Modeling % old C as exponential decay over time yielded a decomposition constant of 0.0325 yr^−1^, considerably lower than decomposition assessed by litterbags (0.22 yr^−1^ for ambient, 0.29 yr^−1^ for elevated). Bars show ± 2 SEM.

**Fig 5:**
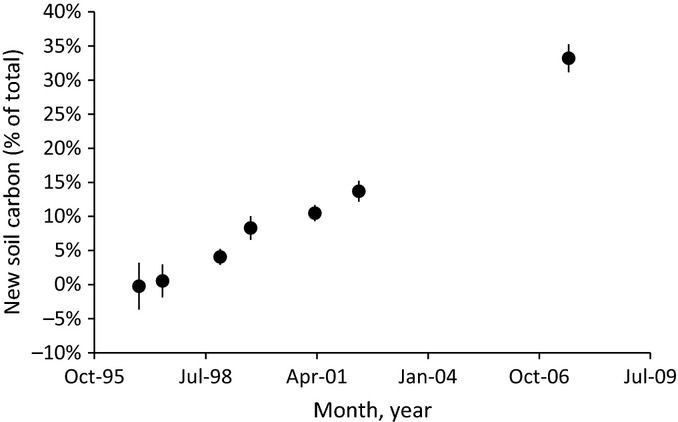
New carbon in surface mineral soils over time. Bars show ± 2 SEM.

**Fig 6:**
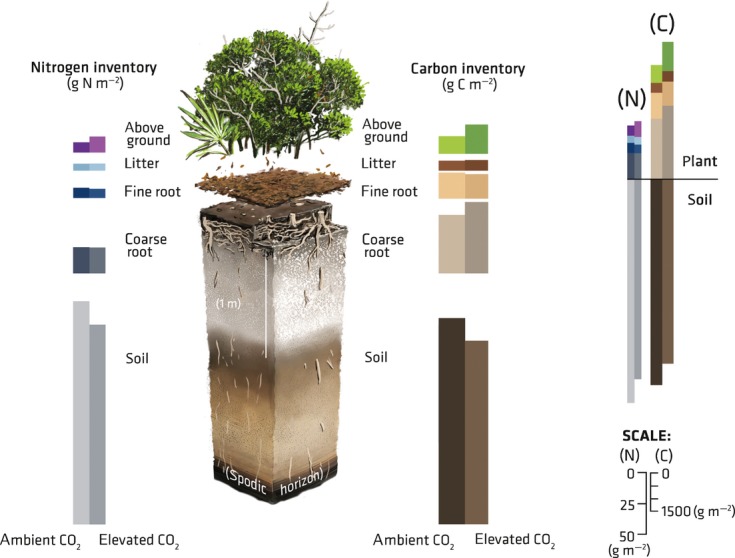
Summary of ecosystem carbon (C) and nitrogen (N) inventories in a subtropical woodland after 11 yr of exposure to elevated CO_2_.

The incorporation of the depleted δ^13^C signature into organic matter pools revealed rates and patterns of flow of ‘new’ C into the system, where new C is that fixed since CO_2_ fumigation began in May 1996. By 2007, coarse roots contained 740 g C m^−2^ of new C, 31% of the total C contained in coarse roots (Fig. 4), yielding a mean C residence time in coarse roots of 35.5 ± 4.2 yr. The total difference in coarse root biomass between E and A was 480 g C m^−2^. This could have been caused entirely by a stimulation of new root C (probably the most parsimonious interpretation), but it is possible that treatments differed in patterns of use of ‘old’, stored C – an idea which should not be immediately dismissed, given that these plants use old C to build new roots (Langley *et al.*, 2002). In the surface soil mineral fraction, the percent new C increased linearly (Fig. 5), with an overall mean residence time of C of 33.6 ± 2.1 yr. In the spodic horizon, there was no evidence of new C accumulation in the total mineral soil or in the light, medium, or heavy density fractions (Table 2). Overall, elevated CO_2_ did not significantly alter the total C content of the system (Table 1), because increased C in plant reservoirs were compensated by reduced C from the soil (Fig. 6).

## Discussion

In this subtropical oak woodland, 11 yr of exposure to elevated CO_2_ increased plant C by 22%, with a smaller (and not significant) effect on plant N of 9%, well within the range of responses typically observed in plants growing under a wide variety of experimental conditions (Norby *et al.*, 2005; de Graaff *et al.*, 2006; Luo *et al.*, 2006). Absolute responses in the mass of C above- and belowground were similar, consistent with elevated CO_2_ having little impact on the partitioning of biomass above- and belowground (Tingey *et al.*, 2000), in contrast to the expectation that root growth would increase disproportionately (Stulen & den Hertog 1993). In our experiment, the relative response aboveground was actually larger than that belowground, because most of the biomass in this system is belowground. The mean residence time of C in coarse roots (revealed by incorporation of the δ^13^C tracer) was sufficiently long that, at the final harvest, only about one third of the C in coarse roots represented new growth over the course of this experiment. By contrast, all of the standing aboveground biomass at the final harvest had accumulated after fire. Thus, repeated cycles of fire disturbance and recovery might yield a larger cumulative response of new C in coarse roots.

The increased C content of plants suggests the potential for elevated CO_2_ to enhance ecosystem C uptake. Yet, increased C contained in plants was not reflected in the C content of soil, neither in the top meter nor in the deeper spodic horizon. Possibly, the experiment lacked sufficient power to detect soil C accumulation (Smith, 2004). Alternatively, other mechanisms may have operated to prevent soil C accumulation in this ecosystem. We can place boundary conditions on the power problem: integrated over the top meter of soil, the mean effect of CO_2_ on total soil C was a decline of −44.3 g C m^−2^ yr^−1^, with the 90% confidence interval spanning a range of CO_2_ effects from more rapid losses of soil C (−132.4 g C m^−2^ yr^−1^) to gain (+ 57.9 g C m^−2^ yr^−1^). This range exhibits the power limitations typical when assessing responses of total soil C to elevated CO_2_ (Hungate *et al.*, 2009). Isolating components of the total soil C reservoir can help overcome the problem of limited power (e.g. Iversen *et al.*, 2012). In our case, we found that by year 6 of the experiment, elevated CO_2_ had reduced the C contained in the light density (Carney *et al.*, 2007) and in the acid-hydrolysable (Langley *et al.*, 2009) fractions of soil C. These findings are consistent with the response we observed at the final harvest reported here where elevated CO_2_ reduced the heavy density fraction of soil C (Table 1) and decreased soluble C susceptible to glucose-induced priming (Fig. 3). The pattern of declining soil C in soil fractions is difficult to reconcile with the concept of soil C accumulation as a first-order response to enhanced plant growth.

The second explanation for not finding soil C accumulation in response to elevated CO_2_ is that it does not occur, because increased C input to soil is compensated by increased C loss. Elevated CO_2_ could enhance export of C through leaching of dissolved organic matter. But, if elevated CO_2_ increased leaching of C in this experiment, this response had no influence on the C content or δ^13^C composition of the spodic horizon; the absence of any effect on δ^13^C is especially unlikely if leaching was an important pathway for C loss. These findings indicate that elevated CO_2_ did not substantially alter leaching losses of C from the system.

In contrast to the absence of any apparent effect on leaching, there was compelling evidence that elevated CO_2_ increased the rate of C cycling through the soil: elevated CO_2_ significantly increased the size and rate of C flow through the labile soil C pool (Fig. 1), it enhanced the proportion of soil C (and N, and ^15^N) that were cycling through the soil microbial biomass (Fig. 2), and it increased the decomposability of labile plant substrates and promoted a physiologically more responsive microbial community (Fig. 3). Elevated CO_2_ also increased fungal biomass, as measured by ergosterol (Klamer *et al.*, 2002), by direct measurements of mycorrhizal fungal biomass (Langley *et al.*, 2003), and by the ratio of fungi to bacteria in the soil microbial biomass, as indicated by the analysis of phospholipid fatty acid profiles (Carney *et al.*, 2007). These results indicate that higher microbial activity was associated with a shift in the composition of the microbial community.

Increased soil microbial activity may also explain why the effect of elevated CO_2_ on the C : N of plant tissues and the litter layer was not apparent, and indeed in some cases may even have been reversed, in soil organic matter. Specifically, elevated CO_2_ increased the C : N ratio of individual plant tissues (Table 3) as commonly observed (Cotrufo *et al.*, 1998; Norby *et al.*, 2001), of the entire plant biomass, above- and belowground, and of the litter layer. Yet, this shift was not observed in soil organic matter after 11 yr of continuous inputs of plant material to the soil organic matter pool. There are two possibilities for this discrepancy: (1) either the inputs of plant material were too low compared to background soil organic matter to drive a change in soil organic C : N; or (2) by increasing soil microbial activity and the processing of C in the soil system, elevated CO_2_ caused a compensatory response, tending to reduce soil C : N. Our finding that elevated CO_2_ reduced the total mass of soil N in the medium density fraction, but increased it in the heavy fraction, is consistent with this second explanation. The medium fraction has a higher C : N ratio than the heavy fraction, and the medium fraction is thought to cycle into the heavy fraction as the soil organic matter is processed by microbial activity and interactions with minerals (Camberdella & Elliott 1992). Thus, the pattern we observe may indicate increased processing and turnover of soil N, promoting transfer to pools with lower C : N ratios, and a tendency for CO_2_ to decrease soil C : N.

Some previous measurements at this site indicated that elevated CO_2_ reduced or had no effect on microbial activity during the first 18 months of the experiment, with reduced gross N mineralization (Hungate *et al.*, 1999) and either reduced or no impact on microbial biomass N (measured as ninhydrin-reactive N) and microbial activity (measured as fluorescein diacetate hydrolysis) in the rhizosphere (Schortemeyer *et al.*, 2000), although the mechanism(s) for these changes were not apparent. These early responses were apparently transient, and did not indicate the decadal-scale response of soil microorganisms to elevated CO_2_. The measurements reported here of microbial biomass, the size of the labile soil C pool, and the distribution and retention of ^15^N cycling through the system are more representative of the entire duration of the experiment (e.g. Fig. 2). Results from this experiment are consistent with the general finding that elevated CO_2_ stimulates soil microbial activity (de Graaff *et al.*, 2006; Dieleman *et al.*, 2012), and the turnover of soil organic matter (Marhan *et al.*, 2010; Phillips *et al.*, 2012; Dawes *et al.*, 2013).

Elevated CO_2_ can stimulate microbial activity by increasing soil water content, especially in grasslands (Hungate *et al.*, 1997a; Morgan *et al.*, 2004), and this response can counterbalance the increased C inputs from enhanced plant growth at elevated CO_2_, causing no change in soil C accumulation (Marhan *et al.*, 2010). In the scrub-oak experiment reported here, elevated CO_2_ slightly increased surface soil water content during the first several years (Hungate *et al.*, 2002), but this effect disappeared with leaf area development (Li *et al.*, 2007), and elevated CO_2_ had no effect on soil temperature (Hymus *et al.*, 2003). Thus, the changes in microbial activity and organic matter turnover that we observed are unlikely to have been driven by differences in temperature, although increased soil moisture may have played a role early on.

Elevated CO_2_ can also increase microbial activity by enhancing the supply of C substrates to soil microorganisms, a response consistent with past reports that, in this experiment, elevated CO_2_ stimulated the ‘priming effect’ (Carney *et al.*, 2007; Langley *et al.*, 2009), the phenomenon where there occurs ‘extra decomposition of native soil organic matter in a soil receiving an organic amendment’ (Bingeman *et al.,* 1953). In the experiment described here, the O_2_ consumption assay indicates that C derived from the litter and roots is more labile in the elevated CO_2_ treatment (Fig. 3), leading to a larger quantity of labile organic matter (Fig. 1). The higher rates of microbial activity observed are consistent with the notion that these new inputs of labile C to soil increased mineralization of native soil organic matter (Van Veen *et al.*, 1991; Carney *et al.*, 2007). This phenomenon has been observed for some time (Lohnis, 1926; Broadbent & Norman, 1947; Broadbent, 1948) and evidence for it has grown: isotope tracer experiments in soil incubations show that substrate additions can more than treble the decomposition rate of native soil organic matter in the short term (Cheng & Johnson, 1998; Cheng *et al.*, 2000). Substrate additions can influence the oxidation of old soil C reservoirs, for example, in deep soil (Fontaine *et al.*, 2007), and can shape the response of soil C to elevated CO_2_ (Hoosbeek, 2004; Trueman & Gonzalez-Meler, 2005; Carney *et al.*, 2007; Hagedorn *et al.*, 2008; Paterson *et al.*, 2008; Taneva & Gonzalez-Meler, 2008; Langley *et al.*, 2009; Trueman *et al.*, 2009; Drake *et al.*, 2011; Reid *et al.*, 2012). Increased oxidation of old soil organic matter is likely a transient response to a change in the rate of labile C inputs. In the experiment described here, the reduction in soil C observed by year 6 (Carney *et al.*, 2007) was comparable to that found after 11 yr, suggesting that the substrates susceptible to priming-induced loss had mostly been degraded during the first 6 yr.

The implications of this response are not limited to C: increased C input to soil, enhancing microbial activity and turnover, can also increasing nutrient availability to plants (Zak *et al.*, 1993). Observations elsewhere that elevated CO_2_ increases microbial activity in concert with greater plant N acquisition from soil are also consistent with this interpretation (Drake *et al.*, 2011), although without direct evidence of increased soil organic matter turnover, increased root exploration is a simpler explanation. Results presented here call into question the notion that feedbacks stimulating soil microbial turnover and N availability necessarily lead to plant N accumulation and increased plant growth. On the one hand, we did find that elevated CO_2_ stimulated plant N uptake and ^15^N dilution in plant tissues, likely driven by increased turnover of soil organic matter mediated by microorganisms (Figs 3; Johnson *et al.*, 1998, 2001; Finzi *et al.*, 2007). On the other hand, increased microbial activity likely promoted N losses, accounting for our finding that elevated CO_2_ reduced recovery of added tracer ^15^N (Table 4).

In this experiment, spanning more than a decade in a naturally occurring ecosystem, photosynthesis and aboveground plant growth exhibited strong responses to chronic exposure to elevated atmospheric CO_2_ (Dijkstra *et al.*, 2002; Seiler *et al.*, 2009), leading to the increased aboveground C content reported here, as well as increased C in coarse roots (Day *et al.*, 2013; Fig. 6). The elevated CO_2_ treatment did not affect C in fine roots at the final harvest, although fine roots responded sporadically in this experiment, with particularly strong responses following the initial fire disturbance and after a hurricane in year 8 (Day *et al.*, 2013). Elevated CO_2_ did not increase soil C, and in fact tended to decrease it, likely a consequence of increased microbial activity. Elevated CO_2_ also increased plant N uptake, possibly driven by higher microbial activity and increased soil N availability, but these responses were also associated with reduced recovery of a long-term ^15^N tracer, likely indicating enhanced ecosystem N losses. Thus, CO_2_ altered the C and N cycles in this ecosystem, but not in ways that promoted large or even detectable increments in total ecosystem C mass. The effect of elevated CO_2_ on soil C turnover via the ‘priming effect’ was large enough to modulate net carbon balance. This finding is not unique, and treatment of this phenomenon in models of soil C cycling is likely warranted (Heimann & Reichstein, 2008; Chapin *et al.*, 2009). While the importance of priming is becoming evident, the challenge to include the phenomenon in models is not trivial: priming is still poorly quantified and the mechanisms remain inscrutable. Meeting this challenge could improve substantially our understanding of terrestrial C cycling, replacing, or at least modifying, the stabilizing first-order kinetics of decomposition used in virtually all current models of the soil C cycle (Luo & Weng, 2011). The response of soil C to labile substrate inputs suggests a previously unrecognized sensitivity of what was thought to be a long-term, stable C sink in the biosphere.

## Supporting Information

Tables S1& S2 Results from ANOVAs testing responses of soil microbial respiration to CO_2_ treatment, habitat, and substrate
