# Supplementary material for: Cumulative response of ecosystem carbon and nitrogen stocks to chronic CO2 exposure in a subtropical oak woodland
Source: New Phytol. 2013 May 30;200(3):753–66. doi: 10.1111/nph.12333 (PMC4282374; doi:10.1111/nph.12333)
Supplement: Tables S1& S2 — Results from ANOVAs testing responses of soil microbial respiration to CO2 treatment, habitat, and substrate [file nph0200-0753-SD1.docx]

| **Supporting Information Tables S1 & S2**  **Table S1** *P*-values from Analysis of Variance testing responses of total O_2_ consumption as affected by habitat (rhizosphere, bulk soil, or litter), CO_2_ (growth CO_2_ condition from which microbial inocula were obtained, ambient or elevated), substrate (root extract versus litter extract), substrate CO_2_ (whether root or litter extracts were obtained ambient or elevated CO_2_ treatments), nutrient additions (N, P), and their interactions. Significant effects indicated by bold type. | | | | |
| --- | --- | --- | --- | --- |
| Effect of: |  | N | P | N × P |
|  |  | **<0.001** | 0.595 | **0.021** |
| Habitat | **<0.001** | 0.279 | **<0.001** | **0.017** |
| Inoculum CO_2_ | 0.776 | 0.878 | 0.259 | 0.865 |
| Substrate | **<0.001** | 0.378 | 0.952 | **0.013** |
| Substrate CO_2_ | 0.175 | **<0.001** | 0.502 | 0.100 |
| Habitat × Inoculum CO_2_ | 0.953 | 0.769 | 0.949 | 0.166 |
| Habitat × Substrate | 0.820 | 0.584 | 0.368 | 0.160 |
| Habitat × Substrate CO2 | 0.148 | 0.359 | 0.705 | 0.690 |
| Inoculum CO_2_ × Substrate | 0.109 | 0.803 | 0.522 | 0.321 |
| Inoculum CO_2_ × Substrate CO_2_ | 0.532 | 0.688 | 0.623 | 0.387 |
| Substrate_CO_2_ × Substrate | 0.102 | 0.310 | 0.647 | 0.408 |
| Habitat × Inoculum CO_2_ × Substrate | 0.366 | 0.789 | 0.163 | 0.587 |
| Habitat × Inoculum CO_2_ × Substrate CO_2_ | 0.880 | 0.830 | 0.156 | 0.505 |
| Habitat × Substrate × Substrate CO_2_ | 0.406 | 0.275 | 0.796 | 0.465 |
| Inoculum CO_2_ × Substrate × Substrate CO_2_ | 0.107 | 0.994 | 0.406 | 0.944 |
| Habitat × Inoculum CO_2_ × Substrate × Substrate CO_2_ | 0.759 | 0.594 | 0.133 | 0.850 |

| **Table S2** *P*-values from 2-way ANOVAs testing for effects of soil habitat and growth CO_2_ concentration from which inocula were collected on the relative responses of substrate utilization (measured as O_2_ consumption) to the addition of glucose, phosphorus, nitrogen, and natural substrates extracted from roots and litter. | | | | | |
| --- | --- | --- | --- | --- | --- |
|  | Root extract | Litter extract | N | P | Glucose |
| Habitat | 0.443 | 0.089 | <0.001 | 0.010 | 0.111 |
| Inoculum CO_2_ | 0.189 | 0.033 | 0.146 | 0.033 | 0.012 |
| Inoculum CO_2_× Habitat | 0.069 | 0.032 | 0.024 | <0.001 | 0.001 |
